# Prevalence of binary toxin positive *Clostridium difficile* in diarrhoeal humans in the absence of epidemic ribotype 027

**DOI:** 10.1371/journal.pone.0187658

**Published:** 2017-11-08

**Authors:** Alan M. McGovern, Grace O. Androga, Daniel R. Knight, Mark W. Watson, Briony Elliott, Niki F. Foster, Barbara J. Chang, Thomas V. Riley

**Affiliations:** 1 School of Biomedical Science, University of Western Australia, Perth, Western Australia, Australia; 2 Department of Microbiology, PathWest Laboratory Medicine, Perth, Western Australia, Australia; 3 Institute for Immunology and Infectious Diseases, Murdoch University, Perth, Western Australia, Australia; 4 School of Medical and Health Sciences, Edith Cowan University, Perth, Western Australia, Australia; 5 School of Veterinary and Life Sciences, Murdoch University, Perth, Western Australia, Australia; Cleveland Clinic, UNITED STATES

## Abstract

Virulence of *Clostridium difficile* is primarily attributed to the large clostridial toxins A and B while the role of binary toxin (CDT) remains unclear. The prevalence of human strains of *C*. *difficile* possessing only CDT genes (A^−^B^−^CDT^+^) is generally low (< 5%), however, this genotype is commonly found in neonatal livestock both in Australia and elsewhere. Zoonotic transmission of *C*. *difficile* has been suggested previously. Most human diagnostic tests will not detect A^−^B^−^CDT^+^ strains of *C*. *difficile* because they focus on detection of toxin A and/or B. We performed a prospective investigation into the prevalence and genetic characteristics of A^−^B^−^CDT^+^ *C*. *difficile* in symptomatic humans. All glutamate dehydrogenase or toxin B gene positive faecal specimens from symptomatic inpatients over 30 days (*n* = 43) were cultured by enrichment, and *C*. *difficile* PCR ribotypes (RTs) and toxin gene profiles determined. From 39 culture-positive specimens, 43 *C*. *difficile* isolates were recovered, including two A^−^B^−^CDT^+^ isolates. This corresponded to an A^−^B^−^CDT^+^ prevalence of 2/35 (5.7%) isolates possessing at least one toxin, 2/10 (20%) A^−^B^−^ isolates, 2/3 CDT^+^ isolates and 1/28 (3.6%) presumed true CDI cases. No link to Australian livestock-associated *C*. *difficile* was found. Neither A^−^B^−^CDT^+^ isolate was the predominant A^−^B^−^CDT^+^ strain found in Australia, RT 033, nor did they belong to toxinotype XI. Previous reports infrequently describe A^−^B^−^CDT^+^ *C*. *difficile* in patients and strain collections but the prevalence of human A^−^B^−^CDT^+^ *C*. *difficile* is rarely investigated. This study highlights the occurrence of A^−^B^−^CDT^+^ strains of *C*. *difficile* in symptomatic patients, warranting further investigations of its role in human infection.

## Introduction

*Clostridium difficile* is an anaerobic, spore-forming gram positive bacillus and a major cause of antibiotic associated life-threatening diarrhoea in humans and animals, particularly pigs. *C*. *difficile* infection (CDI) is the most common healthcare-associated infection in the United States [1]. The classical virulence factors of *C*. *difficile* are the glycosylating large clostridial toxins (LCTs), toxins A and B. These are encoded by the genes *tcdA* and *tcdB*, respectively, located on the LCT pathogenicity locus (PaLoc) [2]. A third toxin, known as *C*. *difficile* binary toxin (CDT), is an actin-specific ADP-ribosyltransferase encoded on a separate pathogenicity island (CdtLoc) [3]. The exact role of CDT in CDI remains unclear. Instances of human infection involving strains of *C*. *difficile* producing only CDT (A^−^B^−^CDT^+^) are infrequent [4–9], yet such strains are often found in animals [10–12]. *C*. *difficile* PCR ribotype (RT) 033 (A^−^B^−^CDT^+^) is the 2^nd^ most prevalent RT in Australian veal calves and piglets [10, 13] while in German calves 62% of isolates were RT 033 or a similar A^−^B^−^CDT^+^ strain, RT 288 [11].

Zoonotic transmission of *C*. *difficile* from animals via food and/or the environment is suggested by overlaps in RTs of *C*. *difficile* found in humans and animals. In Europe and the USA, a virulent lineage of *C*. *difficile* (RT 078) is commonly found in both [14]. The disparity between the prevalence of A^−^B^−^CDT^+^ *C*. *difficile* in humans and animals may be due to differences in methods used for detecting *C*. *difficile*. Human diagnostic laboratories focus on detection of the LCT genes or proteins by nucleic acid amplification or enzyme immunoassay, respectively, while animal studies generally use culture [10–13, 15] due to the poor performance of human diagnostic tests with animal samples [16]. Prior to this study, we had 23 Australian human A^−^B^−^CDT^+^ isolates in our collection, 11 of which (47.8%) were RT 033 (S1 Table), a RT commonly seen in neonatal Australian livestock [10, 13, 15]. This prompted us to investigate the prevalence and molecular epidemiology of human CDI potentially caused by A^−^B^−^CDT^+^ *C*. *difficile* that would otherwise go undetected by conventional diagnostic testing.

## Materials and methods

### Study design

All faecal specimens collected between (and including) 2014/12/31 to 2015/01/29 (30 days) from two of five tertiary public hospitals in Perth, Western Australia (WA), all other public hospitals in the state and certain private laboratories were used for this study. Specimens were submitted as part of routine microbiological investigation, which included specific or reflexive testing for *C*. *difficile*. Each source referred all their routine *C*. *difficile* testing to PathWest Laboratory Medicine, the single public sector pathology service provider for WA. The combined bed capacity of all hospitals in the study was ~3700. Specimens from these referring locations were estimated to account for ~65% of all public *C*. *difficile* testing performed in the state of WA.

All patients included were symptomatic inpatients over 2 years old who had not submitted a stool for CDI testing for more than 8 weeks preceding the study period; thus all CDI cases were considered new [17]. Most samples were also routinely tested for the presence of the enteric pathogens *Salmonella*, *Shigella* and *Campylobacter*. Cases without these organisms but with *C*. *difficile* possessing at least one toxin gene (A, B or CDT) were assumed to be true cases of CDI. If these organisms were detected or only *C*. *difficile* with no toxin genes was recovered, then the case was assumed not to be CDI for the purposes of this study. If testing had not been performed for these organisms, the case was considered indeterminate.

Routine diagnostic *C*. *difficile* testing used the BD MAX^™^ Cdiff assay (BD Diagnostics), a real-time PCR that detects the *tcdB* gene. Only the earliest BD MAX positive specimen from each patient was collected. If no positive specimen existed, the earliest BD MAX negative specimen was used instead such that each patient was represented by one sample. BD MAX negative samples were screened for glutamate dehydrogenase (GDH), a cell wall enzyme common to all strains of *C*. *difficile* regardless of toxin production [18], using the TechLab^®^ CHEK^™^-60 (TechLab) in accordance with the manufacturer’s instructions.

### Identification and isolation of *C*. *difficile*

BD MAX or GDH positive stool samples were inoculated into enrichment broth containing gentamicin (5 mg/L), cycloserine (200 mg/L) and cefoxitin (10 mg/L) supplemented with 0.1% (w/v) sodium taurocholate. After 48 h of incubation, inoculated broths were mixed with an equal volume of absolute ethanol and left at room temperature for 60 min before an aliquot was plated onto chromID^™^ *C*. *difficile* agar (bioMérieux). Incubation of agar plates and identification of *C*. *difficile* was performed as previously described [10]. Co-infection with multiple *C*. *difficile* RTs was analysed by subculturing and PCR ribotyping up to six randomly selected colonies per sample, with priority given to colonies of varying morphology.

### Molecular characterisation of *C*. *difficile*

PCR ribotyping and detection of *tcdA*, *tcdB*, the CDT enzymatic component gene (*cdtA*) and CDT binding component gene (*cdtB*) were performed as previously described [10] with slight modification. The novel primers BE-tcdA-1 (Forward: 5′-CAGTCACTGGATGGAGAATT-3′) and BE-tcdA-2 (Reverse: 5′-AAGGCAATAGCGGTATCAG-3′) specific for the 3’ end of *tcdA* (*tcdA*_3_) were multiplexed with the NK2 and NK3 primers specific for the 5’ end of *tcdA* (*tcdA*_1_) [19]. Reaction mixes (total volume 20 μL) consisted of 4 μL of DNA extract, 10 mM Tris-HCl (pH 8.3) and 50 mM KCl, 1.5 mM MgCl2, 0.2 μM of each primer, 200 μM of each dNTP, 1.25 U AmpliTaq Gold^®^ DNA polymerase and 0.1 mg/mL BSA. Reactions were run with an initial denaturation step of 95 ^o^C for 10 min, followed by 35 cycles of 94 ^o^C for 30 s, 55 ^o^C for 30 s and 72 ^o^C for 90 s, with a final extension step of 72 ^o^C for 7 min. Detection of both *tcdA*_1_ and *tcdA*_3_ fragments was required for an isolate to be considered *tcdA* positive.

Identification of RTs was achieved using the BioNumerics (v7.5, Applied Maths) software package to compare banding patterns with our reference library consisting of 74 reference strains from the UK *Clostridium difficile* ribotyping network (CDRN) and various Australian RTs. Isolates that could not be matched with any reference collection strain were designated with our internal RT prefix “QX”.

For whole genome sequencing (WGS), genomic DNA was extracted from a 48 h blood agar subculture of *C*. *difficile* using the QuickGene Mini80 and QuickGene DNA tissue kit (Kurabo Industries) in conjunction with an MPBio FastPrep-24^™^ 5G (MP Biomedicals) at a speed of 6 m/s for 40 s. Multiplexed paired-end libraries were generated using the KAPA Hyper Prep (KAPA Biosystems). Pooled genomic libraries were sequenced on a MiSeq platform (illumina). Sequence data (trimmed fastq files) have been deposited in the European Nucleotide Archive under study PRJEB19597 (accession ERS1566888–ERS1566897).

Genomes were assembled *de novo* and annotated as previously described [20]. Multilocus sequence type (MLST, ST) was determined *in silico* from assembled contigs using the scheme of Griffiths *et al*. [21]. The presence of toxin genes was determined *in silico* by generating whole genome alignments in Mauve v2.4.0 [22] against *C*. *difficile* reference strain M120 (GenBank accession FN665653). High resolution single nucleotide variant (SNV) analysis was performed on the non-repetitive non-recombinant core genome as previously described [20].

### Ethics statement

As this study used biospecimens that were obtained for clinical purposes and stored by an accredited pathology laboratory no specific human research ethics approval was required under the guidelines set out in the Australian National Health and Medical Research Council (NH&MRC) National Statement on Ethical Conduct in Human Research. Any patient information had been sufficiently anonymised so that neither the patients nor anyone else could identify the patients with certainty.

## Results

### Sample collection, isolation and identification of *C*. *difficile*

**Fig 1:**
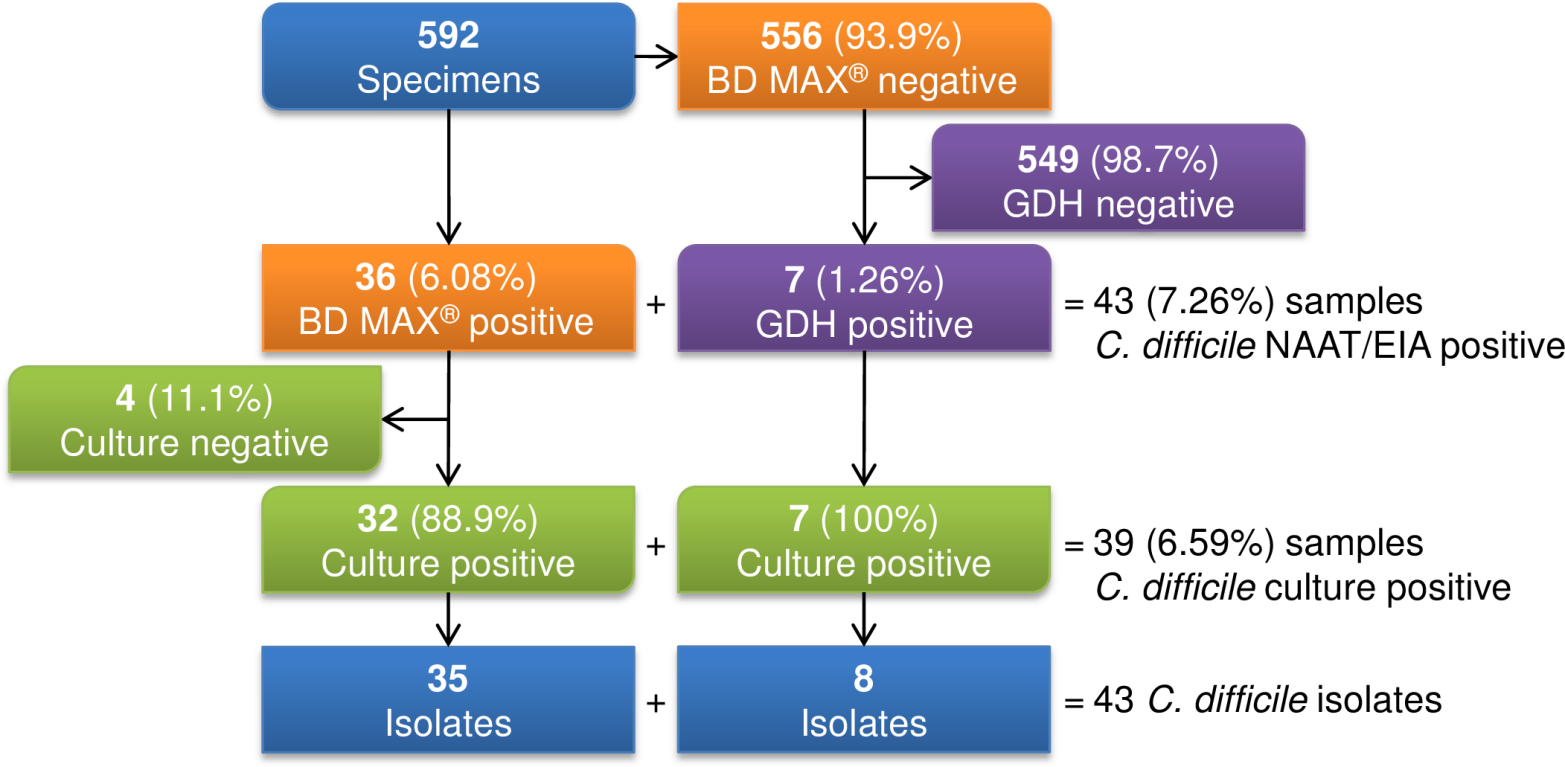
Flowchart of sample collection and isolation. Process flowchart and count of sample collection and *Clostridium difficile* isolation. BD MAX, BD MAX^™^ Cdiff assay (BD diagnostics); GDH, glutamate dehydrogenase.

A sample collection and isolation summary is shown in Fig 1. The number of BD MAX or GDH positive samples was 43 (7.3%) from 592 samples. In total, 43 isolates of *C*. *difficile* were recovered from 39 samples. Of the 39 *C*. *difficile* positive patients, 28 (71.8%) were presumed as CDI, eight (20.5%) as non-CDI and three (7.7%) as indeterminate. Of the 28 patients with CDI, 27 (96.4%) were considered to be ‘typical’ (*tcdB* positive) CDI episodes, with the one remaining case being only CDT positive (3.6%).

### Prevalence and molecular characteristics

Five distinct toxin profiles were identified; the majority of isolates were A^+^B^+^CDT^−^ (*n* = 31, 72.1%) followed by non-toxigenic (A^−^B^−^CDT^−^) (*n* = 8, 18.6%), A^−^B^−^CDT^+^ (*n* = 2, 4.7%), A^−^B^+^CDT^−^ (*n* = 1, 2.3%) and A^+^B^+^CDT^+^ (*n* = 1, 2.3%) strains. Most RTs possessing at least one toxin were RT 014/020 (*n* = 12, 34.3%) followed by RT 012 (*n* = 3, 8.6%) (Table 1). No RT 027 or RT 078 isolates were recovered. The prevalence of CDT^+^ and A^−^B^−^CDT^+^ isolates amongst isolates possessing at least one toxin was 8.6% and 5.7%, respectively (Table 1). A^−^B^−^CDT^+^ isolates comprised two of three CDT^+^ isolates recovered and 20% of A^−^B^−^ isolates (Table 1).

**Table 1:** Ribotype distribution of study isolates.

	PCR Ribotype	Toxin PCR result					PCR Toxin Profile	*n*	% Ribotypes (all)	% Ribotypes (≥ 1 toxin)	% Ribotypes (CDT^+^)	% Ribotypes (A^−^B^−^)
		*tcdA*_1_	*tcdA*_3_	*tcdB*	*cdtA*	*cdtB*						
1	RT 014/020	**+**	**+**	**+**	−	−	A^+^B^+^CDT^−^	12	27.9	34.3		
2	RT 012	**+**	**+**	**+**	−	−	A^+^B^+^CDT^−^	3	6.98	8.57		
3	RT 015	**+**	**+**	**+**	−	−	A^+^B^+^CDT^−^	2	4.65	5.71		
4	RT 056	**+**	**+**	**+**	−	−	A^+^B^+^CDT^−^	2	4.65	5.71		
5	RT 081	**+**	**+**	**+**	−	−	A^+^B^+^CDT^−^	2	4.65	5.71		
6	QX 076	**+**	**+**	**+**	−	−	A^+^B^+^CDT^−^	2	4.65	5.71		
7	RT 001	**+**	**+**	**+**	−	−	A^+^B^+^CDT^−^	1	2.33	2.86		
8	RT 002	**+**	**+**	**+**	−	−	A^+^B^+^CDT^−^	1	2.33	2.86		
9	RT 049	**+**	**+**	**+**	−	−	A^+^B^+^CDT^−^	1	2.33	2.86		
10	RT 053	**+**	**+**	**+**	−	−	A^+^B^+^CDT^−^	1	2.33	2.86		
11	RT 103	**+**	**+**	**+**	−	−	A^+^B^+^CDT^−^	1	2.33	2.86		
12	RT 137	**+**	**+**	**+**	−	−	A^+^B^+^CDT^−^	1	2.33	2.86		
13	QX 001	**+**	**+**	**+**	−	−	A^+^B^+^CDT^−^	1	2.33	2.86		
14	QX 087	**+**	**+**	**+**	−	−	A^+^B^+^CDT^−^	1	2.33	2.86		
15	RT 017	**+**	−	**+**	−	−	A^−^B^+^CDT^−^	1	2.33	2.86		
16	QX 480	**+**	**+**	**+**	**+**	**+**	A^+^B^+^CDT^+^	1	2.33	2.86	33.3	
17	QX 625*	−	−	−	**+**	**+**	A^−^B^−^CDT^+^	1	2.33	2.86	33.3	10
18	QX 626*	−	−	−	**+**	**+**	A^−^B^−^CDT^+^	1	2.33	2.86	33.3	10
19	RT 010	−	−	−	−	−	A^−^B^−^CDT^−^	2	4.65			20
20	RT 051	−	−	−	−	−	A^−^B^−^CDT^−^	2	4.65			20
21	RT 009	−	−	−	−	−	A^−^B^−^CDT^−^	1	2.33			10
22	QX 012	−	−	−	−	−	A^−^B^−^CDT^−^	1	2.33			10
23	QX 121	−	−	−	−	−	A^−^B^−^CDT^−^	1	2.33			10
24	QX 531*	−	−	−	−	−	A^−^B^−^CDT^−^	1	2.33			10
	**Total, *n* (%)**							43	43 (100%)	35 (100%)	3 (100%)	10 (100%)

*tcdA*_1_, toxin A gene 5’ fragment; *tcdA*_3_, toxin A gene 3’ fragment

*new ribotype

Of the three patients with CDT^+^ isolates, two were considered to be true cases of CDI. This included one patient with a specimen positive only for A^+^B^+^CDT^+^ *C*. *difficile* (QX 480) and another patient with an A^−^B^−^CDT^+^ (QX 625) and A^−^B^−^CDT^−^ (QX 531) *C*. *difficile* co-infection. The third patient with A^−^B^−^CDT^+^ *C*. *difficile* (QX 626) was also positive for A^−^B^−^CDT^−^ (RT 051) and A^+^B^+^CDT^−^ *C*. *difficile* (RT 020) but not considered a CDI case due to the concurrent isolation of *Salmonella* Typhimurium. None of the CDT^+^ RTs was a known livestock-associated Australian strain nor could they be matched to our collection of international reference strains. Interestingly, both patients with A^−^B^−^CDT^+^ isolates (AM 0014 and AM 0021) were admitted to the same remote hospital at least 3 days apart. Additionally, the 3’ end of *tcdA* was not detected by PCR in either A^−^B^−^CDT^+^ isolate (Table 1), suggesting they did not belong to toxinotype XI.

The apparent lack of a *tcdA* 3’ fragment, unique RTs and epidemiological clustering of AM 0014 and AM 0021 prompted us to investigate further using WGS. The absence of the entire PaLoc and presence of the complete CdtLoc were confirmed *in silico* for both isolates. Neither isolate belonged to previously known STs, but were single loci variants of various clade 5 lineages. They were given the new ST numbers, ST 392 and 387, respectively (Table 2). SNV analysis conclusively showed that despite epidemiological clustering AM 0014 and AM 0021 were genetically distinct from each other (2104 SNVs) and from 7 other non-toxinotype XI A^−^B^−^CDT^+^ strains in our collection (Table 2).

**Table 2:** Core genome single nucleotide variant analysis of Australian A^−^B^−^CDT^+^ *C*. *difficile* isolates.

PCR Ribotype	ST (Clade)	Toxinotype	PCR Toxin Profile	Host	Origin	Year	Accession	Isolate ID	Pairwise SNV distances^1^									
									RPH 0101	AM 0014^a^	AM 0021^a^	AI 0016^b^	WA 0012^b^	HCD 0052^b^	Q 0006^b^	ES 0548	WA 3103	SE 21C
RT 033	11 (5)	XIa	A^−^B^−^CDT^+^	Human	WA	2007	ERS1566894	RPH 0101		3163	3153	3159	3114	3080	3137	3129	3157	3084
QX 625*	392* (5)	NT	A^−^B^−^CDT^+^	Human	WA	2015	ERS1566889	AM 0014^a^			2104	2206	2707	2497	2722	2812	2124	2685
QX 626*	387* (5)	NT	A^−^B^−^CDT^+^	Human	WA	2015	ERS1566890	AM 0021^a^				2136	2663	2437	2660	2730	936	2669
RT 238	169 (5)	NT	A^−^B^−^CDT^+^	Porcine	WA	2007	ERS1566888	AI 0016^b^					2651	2531	2708	2762	2206	2653
RT 239	168 (5)	NT	A^−^B^−^CDT^+^	Human	WA	2005	ERS1566896	WA 0012^b^						2410	2533	2643	2735	2480
RT 585	164 (5)	NT	A^−^B^−^CDT^+^	Human	WA	1998	ERS1566892	HCD 0052^b^							2327	2525	2475	2350
RT 586	167 (5)	NT	A^−^B^−^CDT^+^	Human	QLD	2007	ERS1566893	Q 0006^b^								2302	2696	2063
QX 143	386 (5)	NT	A^−^B^−^CDT^+^	Human	NSW	2012	ERS1566891	ES 0548									2732	2283
QX 444	169 (5)	NT	A^−^B^−^CDT^+^	Human	WA	2014	ERS1566897	WA 3103										2727
QX 521	280 (5)	NT	A^−^B^−^CDT^+^	Piggery soil	QLD	2015	ERS1566895	SE 21C										

ST, Multilocus sequence type (https://pubmlst.org/cdifficile/); NT, non-toxigenic (i.e. Paloc completely absent)

NSW, New South Wales; QLD, Queensland; WA, Western Australia

^1^lower value indicates closer genetic relatedness

*new ribotype or ST

^a^This study

^b^Elliott *et al*. [6]

## Discussion

Due to previous isolations of suspected Australian livestock-associated A^−^B^−^CDT^+^ *C*. *difficile* in symptomatic patients, we investigated the prevalence of these strains in human CDI, isolating two human A^−^B^−^CDT^+^ *C*. *difficile* strains from 592 faecal samples. Neither isolate was related to the predominant Australian A^−^B^−^CDT^+^ strain of *C*. *difficile*, RT 033, nor were they known to be livestock-associated. Our overall *C*. *difficile* positive rate and RT distribution was similar to that seen previously [23]. Epidemic RTs 027 and 078 were not detected, reflecting the continuing rarity of these RTs in Australia.

Despite recent and increasingly frequent reports of A^−^B^−^CDT^+^ *C*. *difficile* in humans and animals [5–9, 11, 12, 24], the prevalence of these strains in humans is seldom investigated and reports are sporadic [4–9]. Reviews of strain collections, comprising strains from diverse sources collected over many years, indicate that the prevalence of A^−^B^−^CDT^+^ *C*. *difficile* in humans is generally low (< 5%) [4, 8]. A recent study estimated the prevalence of A^−^B^−^CDT^+^ *C*. *difficile* by retrospectively screening 220 A^−^B^−^ consecutively obtained isolates for CDT [5]. These isolates were accumulated from two French metropolitan hospitals over nearly 2 years and Eckert *et al*. [5] recovered one A^−^B^−^CDT^+^ RT 033-like isolate belonging to toxinotype XIb, a prevalence of 0.45% amongst A^−^B^−^ isolates. Our prevalence of A^−^B^−^CDT^+^ *C*. *difficile* amongst A^−^B^−^ isolates was much higher (20%) and observed over a significantly shorter timeframe than Eckert *et al*. [5]. The small sample size of our study, differences in methodology and geographic variation may explain this difference.

PaLoc positive *C*. *difficile* RTs can belong to one of 34 PCR-restriction fragment length polymorphism groups known as toxinotypes [25]. RTs 033 and 288 belong to toxinotype XI, which does not produce the LCTs due to a large deletion leaving only the 3’ end of *tcdA* [25]. Both RT 033 and 288 are ST11 placing them within clade 5 [21]. Of these RTs, RT 033 appears to be the predominant A^−^B^−^CDT^+^ strain in circulation with other toxinotype XI RTs, RT 153 and SLO 187, rarely reported [24, 25]. The absence of RT 033 *C*. *difficile* in our study was not wholly unexpected as nine of 11 (81.8%) previous RT 033 human cases were from outside WA (S1 Table). Additionally, we had previously only isolated RT 033 and RT 288 in Australian animal populations outside WA [10, 13, 15].

All A^−^B^−^CDT^+^ *C*. *difficile* recovered in this study were non-toxinotype XI. Non-toxinotype XI A^−^B^−^CDT^+^ strains are extremely rare; to our knowledge only seven have been described [4, 6]. We previously reported human and animal non-toxinotype XI, A^−^B^−^CDT^+^ *C*. *difficile* in WA [6], yet the A^−^B^−^CDT^+^ RTs encountered in this study were novel. The great heterogeneity of these strains (Table 2) suggests a diverse population of such strains locally. The molecular epidemiology of CDI in Australia appears unique, evidenced by the presence of seemingly exclusive RTs and a diverse population of clade 5 strains [6]. This means our observations may be peculiar to Australia. Conversely, these strains may be distributed globally but remain uncharacterised due to limited adoption of appropriate detection methods in routine surveillance. Recently, diagnostic testing methods have been increasingly incorporating CDT detection [26], usually in order to presumptively identify the A^+^B^+^CDT^+^ RT 027. These tests have the added benefit of potentially detecting A^−^B^−^CDT^+^ *C*. *difficile*. However, as a reflection of the unclear role CDT plays in disease, these tests rarely report CDT specifically and, until recently, did not report A^−^B^−^CDT^+^ results unless prompted to [26].

Both patients with A^−^B^−^CDT^+^ *C*. *difficile* harboured multiple strains of *C*. *difficile* and, in one case, *Salmonella* Typhimurium, possibly suggesting infection from a microbiologically diverse source, such as the environment or food. Community-associated CDI (CA-CDI) is understudied in general and A^−^B^−^CDT^+^ *C*. *difficile* strains are likely to be missed. Additionally, the non-toxigenic *C*. *difficile* present in both our patients may have competed against toxin-producing *C*. *difficile*, protecting the patient in the process [27]. The significance of co-infection in this study and CDI in general remains unclear. Most cases of CDI appear monoclonal in origin with the prevalence of co-infection suggested to be ~10% [28–31]. The simultaneous presence of multiple *C*. *difficile* RTs might indicate an early stage of infection, with one RT yet to dominate others. We could not assess the relative quantities of each co-infecting strain due to our use of enrichment culture. Additionally, it would have been ideal to pick more than 6 colonies from each plate.

An alternative explanation for the low prevalence of animal-associated A^−^B^−^CDT^+^ strains of *C*. *difficile* in humans may be the transient lifecycle of *C*. *difficile* within reservoirs like calves and piglets [10–13]. The prevalence of *C*. *difficile* within these populations rapidly diminishes after three weeks of age [11, 32] unless animals are given antimicrobials directly, or indirectly via the mother. These reservoirs have little link to the general human population beside the slaughter of very young calves and suckling pigs for human consumption, both of which are not widely practised in Australia.

The mild effect of A^−^B^−^CDT^+^ *C*. *difficile* seen recently in hamster and mouse models of infection [33, 34] highlights the need to conclusively prove any epidemiological association between A^−^B^−^CDT^+^ *C*. *difficile* and disease. Such investigations should not be limited to humans, nor should they be limited to symptomatic patients, as asymptomatic patients could also harbour such strains. Future studies will need to comprehensively exclude all alternative causes of CDI symptoms and ideally show symptom resolution due to *C*. *difficile* specific treatment.

To summarise, in a sample of 592 faecal specimens, LCT-negative, binary toxin-positive *C*. *difficile* comprised two of three binary toxin positive human isolates and ~4% of presumed true CDI cases. No link to Australian livestock-associated A^−^B^−^CDT^+^ *C*. *difficile* was established. This study highlights the presence of these strains in symptomatic humans and suggests a diverse population of such strains locally. Larger prevalence surveys and surveillance of animal populations are essential to clarify the relationship between A^−^B^−^CDT^+^ *C*. *difficile* and their human and animal hosts.

## Supporting information

S1 Table Previous human *C*. *difficile* RT 033 A^−^B^−^CDT^+^ isolates. (DOCX)
