# Supplementary material for: Prevalence of binary toxin positive Clostridium difficile in diarrhoeal humans in the absence of epidemic ribotype 027
Source: PLoS One. 2017 Nov 8;12(11):e0187658. doi: 10.1371/journal.pone.0187658 (PMC5678700; doi:10.1371/journal.pone.0187658)
Supplement: S1 Table — (DOCX) [file pone.0187658.s001.docx]

# S1 Table. Previous human *C. difficile* RT 033 A^−^B^−^CDT^+^ isolates.

| **Isolate ID** | **PCR Ribotype** | **ST (Clade)** | **PCR Toxin profile** | **Host** | **Origin** | **Year** |
| --- | --- | --- | --- | --- | --- | --- |
| JIR 8464 | RT 033 | 11 (5) | A^−^B^−^CDT^+^ | Human | VIC | 1982 |
| HCD 0030 | RT 033 | 11 (5) | A^−^B^−^CDT^+^ | Human | VIC | ~1990s |
| JIR 8301 | RT 033 | 11 (5) | A^−^B^−^CDT^+^ | Human | VIC | 2006 |
| JIR 8302 | RT 033 | 11 (5) | A^−^B^−^CDT^+^ | Human | VIC | 2006 |
| JIR 8307 | RT 033 | 11 (5) | A^−^B^−^CDT^+^ | Human | VIC | 2006 |
| JIR 8452 | RT 033 | 11 (5) | A^−^B^−^CDT^+^ | Human | NSW | 2007 |
| JIR 8517 | RT 033 | 11 (5) | A^−^B^−^CDT^+^ | Human | VIC | 2007 |
| RPH 0101 | RT 033 | 11 (5) | A^−^B^−^CDT^+^ | Human | WA | 2007 |
| ES 0561 | RT 033 | 11 (5) | A^−^B^−^CDT^+^ | Human | NSW | 2012 |
| ES 0992 | RT 033 | Unknown | A^−^B^−^CDT^+^ | Human | NSW | Unknown |
| WA 1997 | RT 033 | 11 (5) | A^−^B^−^CDT^+^ | Human | WA | 2013 |
| WA 0012 | RT 239 | 168 (5) | A^−^B^−^CDT^+^ | Human | WA | 2005 |
| ES 0145 | RT 288 | 11 (5) | A^−^B^−^CDT^+^ | Human | NSW | 2006 |
| ES 0940 | RT 288 | Unknown | A^−^B^−^CDT^+^ | Human | NSW | 2012 |
| HCD 0052 | RT 585 | 164 (5) | A^−^B^−^CDT^+^ | Human | WA | 1998 |
| ES 0618 | RT 585 | 164 (5) | A^−^B^−^CDT^+^ | Human | NSW | 2012 |
| ES 0686 | RT 585 | 164 (5) | A^−^B^−^CDT^+^ | Human | NSW | 2012 |
| ES 0896 | RT 585 | 164 (5) | A^−^B^−^CDT^+^ | Human | NSW | 2012 |
| Q 0006 | RT 586 | 167 (5) | A^−^B^−^CDT^+^ | Human | QLD | 2007 |
| ES 0548 | QX 143 | 386 (5) | A^−^B^−^CDT^+^ | Human | NSW | 2012 |
| WA 3103 | QX 444 | 169 (5) | A^−^B^−^CDT^+^ | Human | WA | 2014 |
| ES 0551 | QX 629 | 315 (5) | A^−^B^−^CDT^+^ | Human | NSW | 2012 |
| ES 1173 | QX 635 | 280 (5) | A^−^B^−^CDT^+^ | Human | NSW | Unknown |

ST, Multilocus sequence type (https://pubmlst.org/cdifficile/)

NSW, New South Wales; VIC, Victoria; WA, Western Australia; QLD, Queensland
